# Mouse all-cone retina models of Cav1.4 synaptopathy

**DOI:** 10.3389/fnmol.2023.1155955

**Published:** 2023-04-27

**Authors:** Joseph G. Laird, Ariel Kopel, Colten K. Lankford, Sheila A. Baker

**Affiliations:** Department of Biochemistry and Molecular Biology, University of Iowa, Iowa City, IA, United States

**Keywords:** cone, voltage-gated calcium channel, Cav1.4, α1F, α2δ4, CACNA1F, CACNA2D4, NRL

## Abstract

The voltage-gated calcium channel, Cav1.4 is localized to photoreceptor ribbon synapses and functions both in molecular organization of the synapse and in regulating release of synaptic vesicles. Mutations in Cav1.4 subunits typically present as either incomplete congenital stationary night blindness or a progressive cone-rod dystrophy in humans. We developed a cone-rich mammalian model system to further study how different Cav1.4 mutations affect cones. RPE65 R91W KI; Nrl KO “Conefull” mice were crossed to Cav1.4 α1F or α2δ4 KO mice to generate the “Conefull:α1F KO” and “Conefull:α2δ4 KO” lines. Animals were assessed using a visually guided water maze, electroretinogram (ERG), optical coherence tomography (OCT), and histology. Mice of both sexes and up to six-months of age were used. Conefull: α1F KO mice could not navigate the visually guided water maze, had no b-wave in the ERG, and the developing all-cone outer nuclear layer reorganized into rosettes at the time of eye opening with degeneration progressing to 30% loss by 2-months of age. In comparison, the Conefull: α2δ4 KO mice successfully navigated the visually guided water maze, had a reduced amplitude b-wave ERG, and the development of the all-cone outer nuclear layer appeared normal although progressive degeneration with 10% loss by 2-months of age was observed. In summary, new disease models for studying congenital synaptic diseases due to loss of Cav1.4 function have been created.

## Introduction

Rod and cone photoreceptors are the primary sensory neurons in the visual system. Absorption of light causes graded changes in the membrane potential which is sensed by the voltage-gated calcium channel, Cav1.4, clustered beneath the ribbon that defines the active zone in the synapse. Cav1.4 channels are open at the membrane potential of a dark-adapted photoreceptor and close in response to light. When Cav1.4 is open, the influx of calcium triggers fusion of adjacent synaptic vesicles and release of neurotransmitter. Thus, this channel is essential for communication across the first visual synapse (Lankford et al., 2020; Williams et al., 2022). Cav1.4 additionally participates in development of the synapse as disruption of Cav1.4 coincides with a failure in ribbon elongation and loss or mislocalization of numerous associated proteins (Liu et al., 2013; Zabouri and Haverkamp, 2013; Maddox et al., 2020). In humans, mutations in Cav1.4 subunits result in a spectrum of visual disorders from incomplete stationary night blindness (CSNB2) to progressive cone-rod dystrophy (Hauke et al., 2013; Michalakis et al., 2014; De Silva et al., 2021; Du et al., 2022).

It is not known why Cav1.4 mutations present as different diseases. Cav1.4 is composed of three subunits: α1F is the largest, spans the membrane and forms the pore, β2a is found on the intracellular side of the channel and participates in the trafficking of the channel and interactions with synaptic ribbon-associated proteins, α2δ4 is found on the extracellular side of the channel and is thought to influence the stability of the channel and interact with a trans-synaptic adhesion protein (reviewed in 9). In humans, most of the reported mutations in CACNA1F (encoding α1F) are diagnosed as CSNB2 (De Silva et al., 2021). This implies α1F dysfunction is predominantly a rod disease. However, many patients do not have night blindness leading to the use of the alternative names cone-rod synaptic disorder or congenital stationary synaptic dysfunction (Boycott et al., 2000; Littink et al., 2009; Bijveld et al., 2013; Stone et al., 2017). Some people with CACNA1F mutations have been diagnosed with cone-rod dystrophy (Jalkanen et al., 2006; Hauke et al., 2013; Du et al., 2022). The range of retinal dysfunction associated with CACNA1F mutations is consistent with mouse models where both gain-of-function and loss-of-function mutations impair rod and cone synapses (Mansergh et al., 2005; Chang et al., 2006; Doering et al., 2008; Specht et al., 2009; Knoflach et al., 2013; Regus-Leidig et al., 2014; Dai et al., 2019).

There is a seeming discrepancy regarding the α2δ4 subunit. Analysis of two independent α2δ4 KO mouse lines came to the same conclusion, that presynaptic structure and function are much more severely disrupted in rods compared to cones (Wycisk et al., 2006; Wang et al., 2017; Kerov et al., 2018). Whereas humans with mutations in CACNA2D4 (encoding α2δ4) cause a cone-dominant disease (RCD4, Wycisk et al., 2006; Ba-Abbad et al., 2016). One explanation is that disease mutations in CACNA2D4 are so rare that the available patient information does not represent the spectrum of possible disease. Alternatively, in the mouse models the dramatic defects in rod synapses and relative paucity of cones [180,000 cones compared to 6.4 million rods (Jeon et al., 1998)] could have led to a subtle phenotype being overlooked. This consideration prompted us to develop a cone-rich mammalian model system for studying Cav1.4.

The Nrl KO mouse is frequently used to make it easier to study mammalian cones because the transcription factor that is required for specification of rods is lacking thus, rod precursors develop into S-cone-like cells, creating an all-cone retina (Mears et al., 2001; Daniele et al., 2005). Despite the advantage of a murine all-cone retina, the Nrl KO can be difficult to work with for developmental studies because of the retinal infoldings or neural rosettes that form (Stuck et al., 2012). This problem is abrogated in a double-mutant strain where the Nrl KO was crossed to a strain with reduced 11-cis retinal levels, the RPE65 R91W knockin (Samardzija et al., 2014), which we call “Conefull” to more simply distinguish it from the Nrl KO parental strain. Conefull mice have an anatomically normally developing retina containing only cone photoreceptors. In this study, we crossed two Cav1.4 subunit mutant strains onto the Conefull background to further investigate the role of Cav1.4 in the development and function of cone synapses.

## Materials and methods

### Animals

C57BL/6 J (RRID:*IMSR_JAX:000664*) were used as wildtype (WT) controls. Cav1.4 α1F KO (MGI:38.8717, RRID:*IMSR_JAX:017761*) and α2δ4 KO mice (MGI:6200657, RRID: *IMSR_JAX:035183*) have been previously described (Specht et al., 2009; Kerov et al., 2018). RPE65 R91W KI; Nrl KO (Conefull) mice were a generous gift from Christian Grimm (Samardzija et al., 2014); Conefull:α1F KO and Conefull:α2δ4 KO mice were generated for this study by crossing Conefull mice to α1F KO or α2δ4 KO, respectively. Animals were genotyping using published PCR protocols or through the services of Transnetyx (Cordova, TN). Mice of both sexes, up to the age of 6 months were used. Mice were housed in a central vivarium, maintained on a standard 12/12-h light/dark cycle, with food and water provided *ad libitum* in accordance with the Guide for the Care and Use of Laboratory Animals of the National Institutes of Health. All procedures adhered to the ARVO Statement for the Use of Animals in Ophthalmic and Vision Research and were approved by the University of Iowa IACUC committee.

### Visually guided water maze

Mice were trained to swim under ambient room lighting in a 4-foot diameter pool to a 4-inch diameter, high contrast, visible escape platform. After training, a series of 30 test trials over 6 days were conducted under photopic conditions (luminance of 11.1 cd/m^2^), and after overnight dark adaptation under scotopic conditions (luminance of 0.002 cd/m^2^) as previously described (Kerov et al., 2018).

### Electroretinography

All ERG recordings were obtained on the Espion V6 Diagnosys Celeris system (Diagnosys LLC, Massachusetts). Mice were dark-adapted overnight, and dim red lighting used for all subsequent steps. Mice were anesthetized with a mixture of ketamine (87.5 mg/kg) and xylazine (2.5 mg/kg); body temperature was maintained by keeping animals on a heating pad. Tropicamide (1%) was used to dilate the pupils and Genteal gel (0.3% Hypromellose) was used to keep the eyes hydrated. Animals were light adapted for 10 min and background illumination (1.5 log cd.s/m^2^) was maintained as the responses to a series of increasingly bright test flashes (−0.6, 0.0, 0.4, 0.9, 1.4, 1.9, 2.4, 2.9 log cd.s/m^2^) were recorded. Recordings were collected from both left and right eyes. The a-wave amplitude was measured as the amplitude from the baseline to the first negative trough for the brightest flash only (2.9 log cd.s/m^2^). Mice ranged in age from 1 to 2 months and six animals of each genotype were tested.

### Optical coherence tomography

Mice were anesthetized with ketamine/xylazine and tropicamide (1%) was used to dilate the pupils. Images were collected with a Bioptigen spectral-domain imaging system (Bioptigen, Inc.) equipped with a mouse retina objective, reference arm position set at 1264. Scan parameters were as follows: rectangular (1.4 mm^2^) volume scans, 1,000 A-scans/B-scan, 33 B-scans/volume, 3 frames/B-scan, and 1 volume. To quantify degeneration rates, the distance between the RPE and NFL bands was measured using Photoshop CC (Adobe) with calibration to the vertical scale bar in 4–8 images adjacent to the optic nerve for each animal; 4–6 animals were used for each genotype at each age.

### Antibodies

Rabbit polyclonal antibodies against the α1F or α2δ4 Cav1.4 subunit were a gift from Amy Lee (Liu et al., 2013; Kerov et al., 2018) and used at a dilution of 1:1000 for Western blotting. Antibodies used for immunohistochemistry were: SV2B (Synaptic Systems, Cat # 119102, RRID: AB_887803, diluted 1:1000), CTBP2 (BD Biosciences, Cat # 612044, RRID:AB_399431, diluted 1:500), PKC alpha (Santa Cruz, Cat # sc-208, RRID:AB_2168668, diluted 1:500), and secretagogin (BioVendor, Cat # RD181120100, RRID:AB_2034060, diluted 1:1000). Secondary anti-rabbit and anti-mouse antibodies conjugated to Alexa 488 or 594 were obtained from Thermo Fisher.

### Western blotting

Membrane fractions of retina were prepared by homogenization of 8 retina in 1 ml of hypotonic buffer (50 mM Tris–HCl, 10 mM NaCl, 0.32 M sucrose, 5 mM EDTA, 2.5 mM EGTA, pH7.4, supplemented with a protease inhibitor cocktail tablet) and clarified by centrifugation at 2000 x g for 10 min. Membranes were pelleted by centrifugation at 240,000 × g for 30 min, the supernatant was discarded and 0.4 ml of hypotonic buffer supplemented with 1% DDM was added to the pellet; after incubation on ice for 30 min followed by vortexing, the resolubilized membranes were mixed with reducing LDS sample loading buffer and 15–30 μL was run on SDS-PAGE gels for Western blotting as previously described (Inamdar et al., 2021).

### Immunohistochemistry

Immunostaining was carried out as previously described (Pan et al., 2014; Kerov et al., 2018). Briefly, eyes (for animals less than postnatal day 8) or posterior eyecups were collected by dissection, fixed in 4% paraformaldehyde at room temperature for 15–45 min, cryoprotected in 30% sucrose, and then frozen in O.C.T. (Tissue-Tek, Electron Microscopy Sciences, Hatfield, PA). Radial sections were cut and collected on electrostatically charged glass slides, and either labeled immediately or stored at −80°C until use. Blocking buffer consisted of 10% normal goat serum and 0.5% Triton X-100 in PBS. Primary and secondary antibodies (diluted in blocking buffer) were incubated on retinal sections for 1–3 h at room temperature or overnight at 4°C. Images were acquired on a THUNDER Imager (Leica DM6B microscope equipped with a Leica DFC9000 GT camera). Computational clearing of z-stacks was performed using LASx software.

### Quantitative image analysis

Retinal thickness in OCT images was measured in Adobe Photoshop by measuring the length of at least 10 lines per image drawn perpendicular to the retina from the RPE to the NFL. At least 5 images/animal and 6 animals/genotype were analyzed. Histology images were collected using a THUNDER Imager (Leica DM6B microscope equipped with a Leica DFC9000 GT camera). The area of the developing nuclear-free OPL was measured in Adobe Photoshop using the magnetic lasso tool to capture the irregular area. At least 3 eyes/animal/genotype were measured.

### Statistical analysis

Statistical differences were determined using GraphPad Prism software (v8). Statistical significance was defined using an alpha of 0.05. ANOVA with Dunnett’s multiple comparisons were used. In the text mean is reported with the SEM, in all graphs the variability is shown by plotting mean ± S.D.

## Results

### Cav1.4 mutants in a cone-only retina

**Figure 1:**
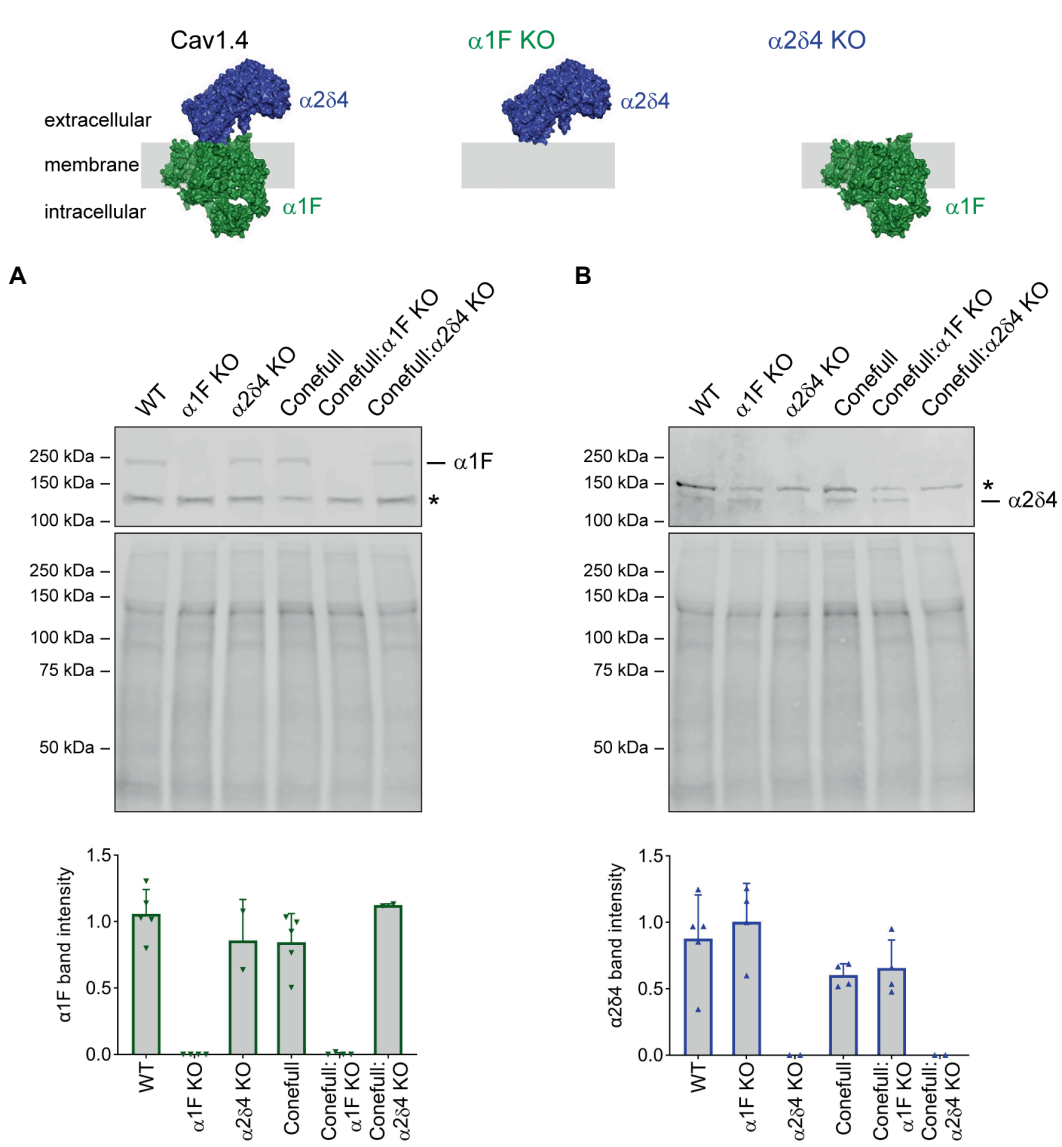
Validating loss of the respective Cav1.4 subunits in the new Conefull strains. Cartoons of Cav1.4 illustrating the knockouts used in this study. The cartoons were drawn using the structure of Cav1.3 (PDB ID 7UHG) as reference. **(A)** Western blot for α1F, specific band is at 230 kDa, the asterisk indicates a non-specific signal. Mid-panel is total protein stain used for normalization as shown in the lower graph, bars are S.D. **(B)** Western blot for α2δ4, specific band is at ~130 kDa, the asterisk indicates a non-specific signal. Mid-panel is total protein stain used for normalization as shown in the lower graph, bars are S.D.

The RPE65 R91W KI; Nrl KO strain, referred to as “Conefull,” was crossed with two different Cav1.4 mutants: α1F KO to generate “Conefull:α1F KO,” or α2δ4 KO to generate “Conefull:α2δ4 KO.” Cav1.4 is expressed at relatively low abundance, so retina was separated into soluble and membrane fractions and the membrane fraction was used for Western blotting to confirm the loss of the respective Cav1.4 subunit. Expression of α1F was absent in the α1F KO and Conefull:α1F KO strains, while α2δ4 was preserved in these samples (Figure 1A). Expression of α2δ4 was absent in the α2δ4 KO and Conefull:α2δ4 KO strains, while α1F was preserved (Figure 1B).

### Assessment of adult Conefull: Cav1.4 KOs

**Figure 2:**
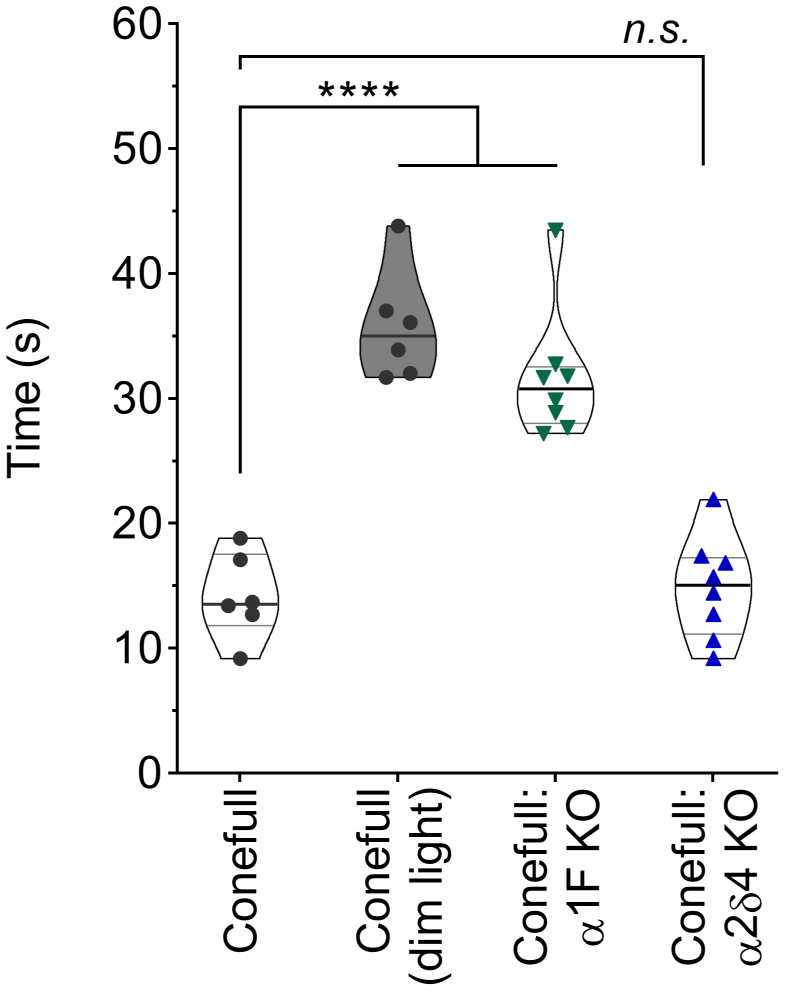
Vision dependent navigation is defective in Conefull: α1F KO. Time to reach the visible escape platform for Conefull animals under photopic (black circles) or scotopic (black circles, shaded violin plot) lighting compared to Conefull: α1F KO (green triangles) or Conefull: α2δ4 KO (blue triangles) under photopic lighting.

We examined the function and structure of the retina in adult Conefull: Cav1.4 KOs and began with a visually guided water maze to assess visual behavior (Specht et al., 2009; Kerov et al., 2018). Conefull mice completed the task under photopic conditions (average room lighting) with a group average of 14.2 ± 1.4 s. Under scotopic conditions (dim lighting) that requires the sensitivity of rod-mediated vision, Conefull mice required 35.8 ± 1.8 s to complete the task. This condition serves as the positive control for impaired vision (Figure 2; Table 1; Laird et al., 2019). The Conefull:Cav1.4 KOs were only tested under photopic conditions. Conefull:α1F KO required 31.7 ± 1.8 s to complete the task, which is not significantly different than the scotopic Conefull control, while Conefull:α2δ4 KO animals were not impaired as they completed the task in 14.8 ± 1.4 s. This differing impact on behavioral vison due to the loss of the different Cav1.4 subunits is the same as observed in the rod-dominated wildtype background (Wang et al., 2017; Kerov et al., 2018).

**Table 1:** Statistical description of visually guided water maze.

					Mean rate differences compared to Conefull	ANOVA + Dunnett’s, adj *p* value
Genotype	Lighting	Age (mon)	*N*	Mean ± SEM (s)		
Conefull	Photopic	2.6–4.8	6	14.2 ± 1.4	n/a	
Conefull	Scotopic	0.9–1.3	6	35.8 ± 1.8	Δ21.6 s, 95% CI [15.1,28.1]	<0.0001
Conefull: α1F KO	Photopic	2.0–2.9	8	31.7 ± 1.8	Δ17.5 s, 95% CI [11.5,23.6]	<0.0001
Conefull: α2δ4 KO	Photopic	1.3–2.0	8	14.8 ± 1.4	Δ0.7 s, 95% CI [−5.2, 6.6]	0.9819

**Figure 3:**
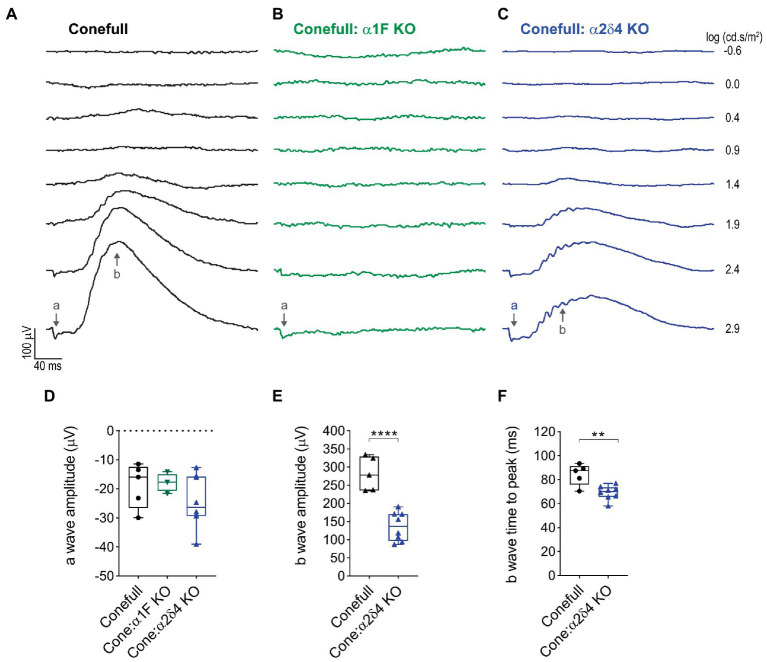
The no b-wave phenotype is present in Conefull: α1F KO. **(A)** Flashes of increasing light intensity evoked a positive going b-wave response in Conefull animals beginning at 1.4 log (cd.s/m2) consistent with absence of rod function. **(B)** An a-wave but not a b-wave was elicited in Conefull: α1F KO, green traces. **(C)** Both an a-wave and b-wave was elicited in Conefull: α2δ4 KO, blue traces. **(D)** Comparison of a-wave amplitude, **(E)** b-wave amplitude and **(F)** b-wave time to peak in response to 2.9 log (cd.s/m2) flash intensity.

Photopic flash ERG was used to test retinal function. As expected, responses were elicited only from brighter flashes (> 1.4 log cd.s/m^2^) that activate cones. The a-wave which is due to activation of the phototransduction cascade was present and not different in the Conefull: Cav1.4 subunit mutants. The b-wave which is largely due to synaptic transmission from photoreceptors to ON-bipolar neurons was absent from Conefull: α1F KO. In Conefull: α2δ4 KO mice the b-wave was present but reduced in amplitude (Figure 3; Table 2). The time to peak for the b-wave was slightly accelerated. The significance of this is not clear, especially since oscillatory potentials that overlap the rising b-wave were more apparent in the traces from Conefull: α2δ4 KO compared to Conefull. Overall, the ERG findings are consistent with the reports from the respective KOs in the rod-dominant wildtype background (Mansergh et al., 2005; Specht et al., 2009; Wang et al., 2017; Kerov et al., 2018).

**Table 2:** Statistical description of ERG response to 2.9 log (cd.s/m^2^) flash on saturating background.

Genotype	*N*	Parameter	Mean ± SEM	Mean differences (compared to Conefull)	ANOVA + Dunnett’s, adj *p* value
Conefull	5	a-wave amplitude	−19 ± 3 μV	n/a	n/a
		b-wave amplitude	282 ± 21 μV	n/a	n/a
		b-wave time to peak	84 ± 4 ms	n/a	n/a
Conefull: α1F KO	4	a-wave amplitude	−18 ± 2 μV	Δ1, 95% CI [−14,13]	0.9730
		b-wave amplitude	Not Measurable	n/a	n/a
		b-wave time to peak	Not Measurable	n/a	n/a
Conefull: α2δ4 KO	8	a-wave amplitude	−24 ± 3 μV	Δ5.5, 95% CI [−5,16]	0.3620
		b-wave amplitude	137 ± 14 μV	Δ145, 95% CI [92,198]	< 0.0001
		b-wave time to peak	69 ± 2 ms	Δ15, 95% CI (Wycisk et al., 2006; Michalakis et al., 2014)	0.0015

**Figure 4:**
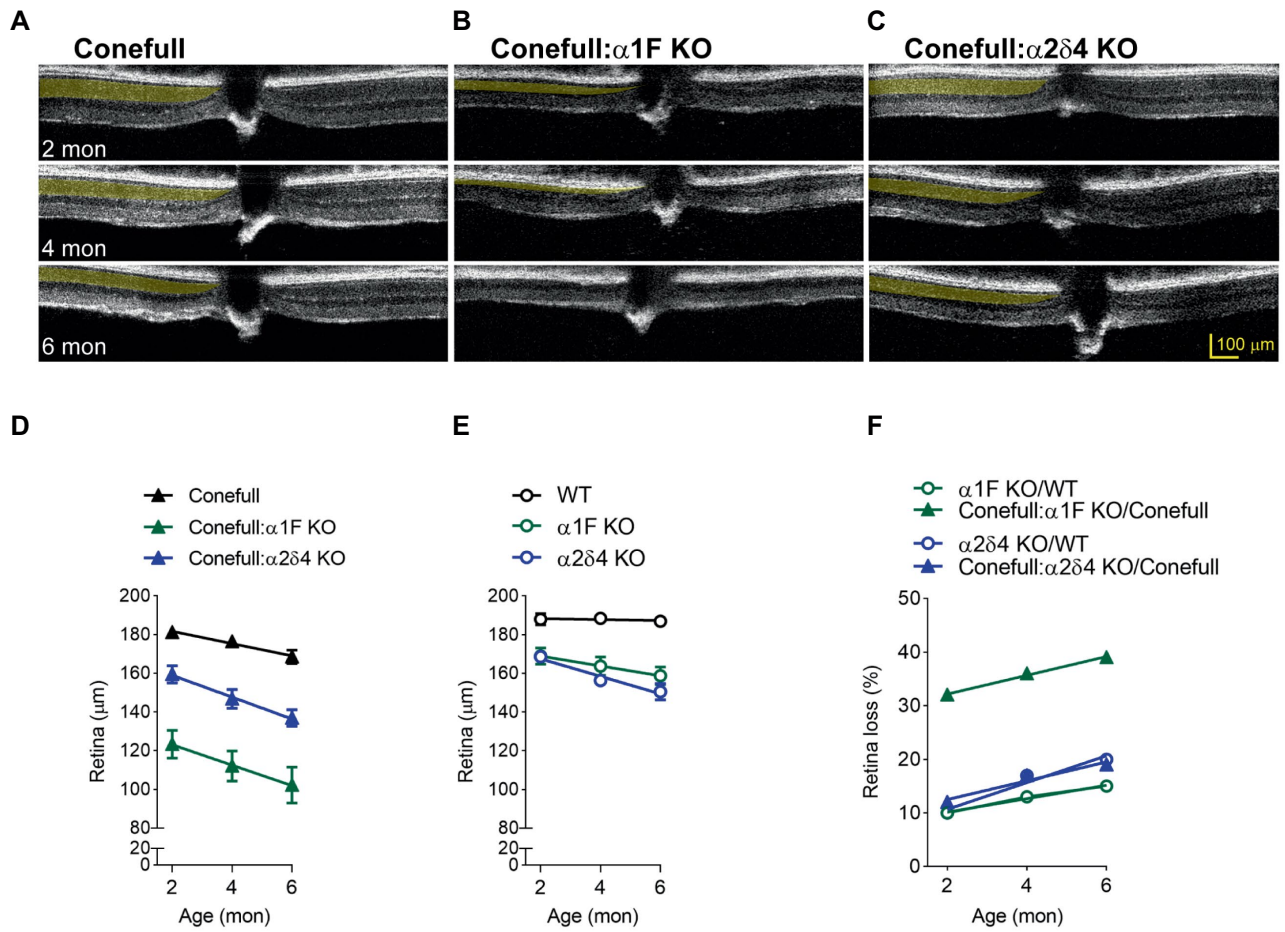
Retinal degeneration is severe in Conefull: α1F KO. Representative OCT images of **(A)** Conefull, **(B)** Conefull: α1F KO, or **(C)** Conefull: α2δ4 KO with the photoreceptor layer to the left of the optic nerve highlighted in yellow at 2, 4, or 6 months of age. **(D)** Retinal thickness from 2 to 6 months for Conefull (black), Conefull: α1F KO (green), or Conefull: α2δ4 KO (blue). **(E)** Retina thickness from 2 to 6 months for wildtype, α1F KO (green), or α2δ4 KO (blue). **(F)** Retinal loss normalized to strain background. Degeneration due to α1F KO (green) is more severe in the Conefull background whereas degeneration in α2δ4 KO (blue) is not.

OCT was used to assess gross retina anatomy. Previously, we have analyzed mouse OCT images by measuring the thickness of the outer nuclear layer (ONL), from the outer plexiform layer (OPL) to the outer limiting membrane (OLM; Kerov et al., 2018). However, in the Conefull retina the OLM is not readily distinguished so we analyzed this data by measuring full retina thickness, from the nerve fiber layer (NFL) to the first of the hyperreflective lines originating from the RPE-Choroid. In OCT images from animals aged 2–6 months the conefull retina thinned from 181.2 ± 2.6 μm to 176.4 ± 2.5 μm at a rate of 3.2 ± 0.5 μm/mon (Figures 4A,D). In comparison to WT retina, the conefull retina is thinner by 4 to 10% over this age range (consistent with the previously described degeneration occurring in this strain (Samardzija et al., 2014). The retinas of Conefull:α1F KO were thinner than Conefull at all ages examined by OCT. As Conefull:α1F KO animals aged from 2 to 6 months, the retina thinned from 123.3 ± 7.2 μm to 102.2 ± 9.2 μm at a rate of 5.3 ± 0.2 μm/mon (Figures 4B,D). The retinas of Conefull: α2δ4 KO mice also progressively thinned from 159.3 ± 4.5 μm to 136.9 ± 4.3 μm at a rate of 5.6 ± 0.4 μm/mon (Figures 4C,D). Thus, there is degeneration in the Conefull: Cav1.4 KOs above the levels seen in Conefull.

To provide context, we measured the full retina thickness in OCT images from a cohort of α1F KO and α2δ4 KO since they undergo a mild degeneration (Figure 4E; Regus-Leidig et al., 2014; Kerov et al., 2018). After normalizing to the different background strains, it is easier to see that the loss of the α1F subunit has a larger effect in the Conefull background with thinning over 2–6 months progressing from 32–49%, but only 10–15% in the rod-dominant WT background (Figure 4F). However, the rate was not different indicating that the significant thinning of the Conefull: α1F KO at 2-month of age could be due more to problems with development than maintenance. The loss of the α2δ4 subunit resulted in similar retinal thinning in both strains; 12–19% for the Conefull background versus 10–20% for the WT background (Figure 4F). In conclusion, this set of experiments revealed a larger than expected effect due to loss of α1F while the effect due to loss of the α2δ4 subunit is milder.

### Lamination defects in developing Conefull: Cav1.4 KOs

**Figure 5:**
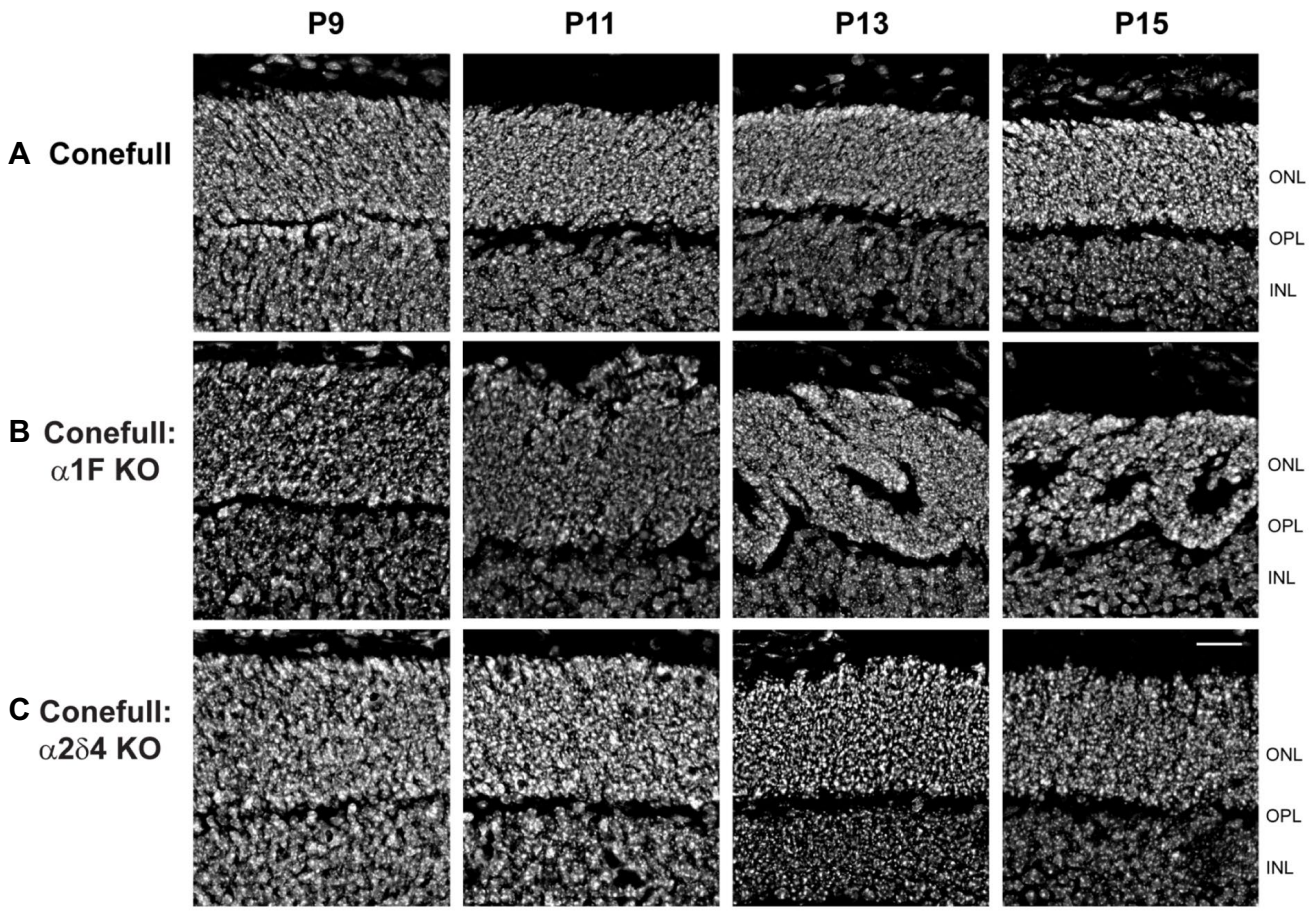
Neural rosettes form in Conefull: α1F KO as cones mature. Retinal cross-sections showing the outer and inner nuclear layers labeled with Hoechst from **(A)** Conefull, **(B)** Conefull: α1F KO, or **(C)** Conefull: α2δ4 KO at ages P9 - P15. Scale bar is 20 μm.

**Figure 6:**
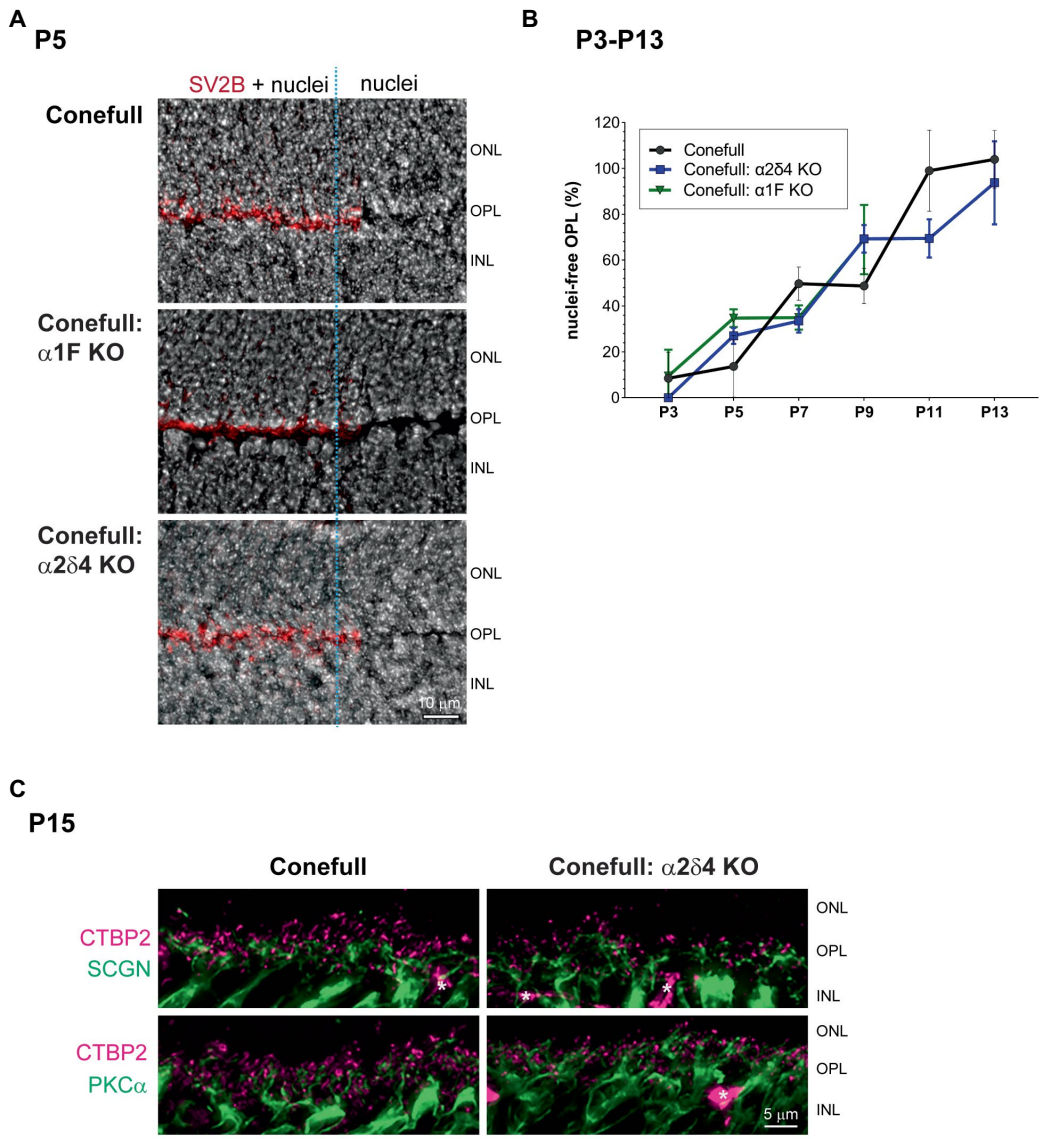
Development of the OPL is similar in Conefull and Conefull: Cav1.4 mutants. **(A)** Representative Hoechst stained P5 retinas of each genotype labeled with SV2B (red) to mark the presynaptic terminal in the developing OPL. The right 1/3 of each image (marked with the blue dashed line) has the SV2B channel removed to clearly show the nuclei-free OPL. Scale bar is 10 μm. **(B)** The area (mean ± S.D.) of the nuclear-free zone between the developing ONL and INL was measured from images as shown in Figure 5 and **A** then plotted as a function of animal age from P3-P13 for Conefull and Conefull: α2δ4 KO (blue line). Conefull: α1F KO (green line) could only be measured from P3-P9 because retina morphology was disrupted by neural rosettes at the later timepoints. **(C)** Comparison of synapses in the OPL at P15 in Conefull compared to Conefull: α2δ4 KO retina. Presynaptic ribbons labeled with CTBP2 (magenta) and bipolar cell dendrites (green) with secretagogin (SCGN) for a subset of cone ON and OFF bipolar cells in the upper panels or PKCα for rod ON bipolar cells in the lower panels. Asterisks indicate non-specific labeling of blood vessels by the secondary antibody, scale bar is 5 μm.

Since retina loss in Conefull: α1F KO is already striking at 2 months of age we set out to test when degeneration began. We examined retinas from postnatal day 7 (P7) to P15 because that brackets the time of cone maturation and eye opening (Figure 5). At P7 the Conefull: α1F KO retina was normal, but at P9 isolated neural rosettes in the ONL were occasionally observed. The number of rosettes increased in number and size so that by P11 at least half of the ONL was completely rosetted. These structures may form as the retina reorganizes to compensate for cone cell dropout. Rosettes did not form in either Conefull or Conefull: α2δ4 KO (Figure 5). We also examined retinas earlier in developmental time (P3-13). Retinas were immunolabeled with the synaptic vesicle marker, SV2B to assist in identifying the forming OPL (representative images from P5, Figure 6A). As synapses form and the OPL matures nuclei migrate out leaving a nuclei-free zone. We measured this area in all three genotypes, note the Conefull: α1F KO retina was only analyzed between ages P3-P9 to avoid interference from the rosettes. We found that lamination proceeded the same in all three genotypes (Figure 6B). Despite the normal area of the OPL, ribbon density appeared decreased in Conefull: α2δ4 KO retina at P15 although the ribbons were positioned appropriately at the tips of bipolar dendrites (Figure 6C). Altogether this work provides a characterization of new mouse models for investigating Cav1.4-dependent synapse development and signaling in an all-cone mouse retina.

## Discussion

Our findings regarding visual function of Conefull: α1F KO mice are well aligned with previous studies in related models. The Conefull: α1F KO mice lacked the ability to navigate a visually guided water maze, a commonly used test of mouse vision. ERG recordings had normal a-waves but there was no b-wave consistent with the expectation that cones maintain the ability to respond to light normally but synaptic function at the cone to ON-bipolar ribbon synapse was lost. Mild progressive retinal degeneration occurs in α1F KO mice, but the more striking morphological feature is that in the absence of Cav1.4 the outer plexiform layer is reduced in thickness by half consistent with defective development of the presynaptic terminal (Mansergh et al., 2005; Chang et al., 2006; Specht et al., 2009; Regus-Leidig et al., 2014; Dai et al., 2019). The abnormal thinness of the OPL is a feature shared by α1F mutant rats and larval zebrafish, but it is not clear if that leads to retinal degeneration in those models (Gu et al., 2008; Schlegel et al., 2019). We were therefore surprised to find comparatively aggressive retinal degeneration in Conefull: α1F KO mice. Taking into consideration the retinal degeneration that occurs in Conefull animals, we observed 30% retina loss in Conefull: α1F KO compared to only 10% loss in α1F KO mice at 2-months of age. Retinal morphology of Conefull: α1F KO mice was normal through post-natal development until the time of eye opening (~P13) when rosettes in the ONL became a consistent phenotype. Rosettes are a characteristic of the Nrl KO retina and have been attributed to disorganization of the outer limiting membrane (an array of adherens junctions between Muller glia and photoreceptors) and delayed maturation of some photoreceptors (Stuck et al., 2012). While rosettes do not form in Conefull retina, we do not see a hyperreflective band in the OCT that would correspond to the position of a well-organized outer limiting membrane. We speculate that rosettes appear in Conefull: α1F KO because when the eyelids open and there is increased light, cones start to die. The loss of cells from the ONL in combination with the disorganized outer limiting membrane causes remodeling of the ONL. We conclude that Conefull: α1F KO mice are useful for further comparative exploration of mammalian cone synapses but only early in development.

The loss of α2δ4 in normal rod-dominant mice has more dramatic effects on rod synaptic structure and function than on cones. In line with this, the Conefull: α2δ4 KO mice had sufficient vision to navigate the water maze. The ERG had an intact a-wave with a reduced and slightly accelerated b-wave. We also noted a slightly decreased density of ribbons in Conefull: α2δ4 at P15 which could explain the reduced amplitude of the b-wave. Consistent with the milder functional phenotype, degeneration occurs in Conefull: α2δ4 KO, but at a degree comparable to normal rod-dominant mouse retinas lacking α2δ4. As in the Conefull: α1F KO, we did not see clear thinning of the OPL, in both cases this may be due to changes in the shape and size of cone terminals observed in the Nrl KO at the ultrastructural level (Strettoi et al., 2004). The α2δ4 subunit is required to stabilize expression of the pore-forming subunit (Wang et al., 2017; Kerov et al., 2018). Note, that in our western blots we observed expression of α1F which was not seen before but that is likely because here we first enriched for membranes instead of using whole retina lysates perhaps allowing us to detect protein below the sensitivity limit of the antibodies. We could not determine if the α1F was in the presynaptic membrane or internal biosynthetic membranes. The α2δ4 subunit also contributes to regulating the gating properties of Cav1.4 and can link the channel to transsynaptic adhesion molecules. Despite these overlapping functions our work affirms that loss of α2δ4 is less disruptive than loss of α1F.

The question as to why humans with mutations in CACNA2D4 have a cone-rod dystrophy remains. Our analysis of the Conefull: α2δ4 KO indicates the discrepancy is not due to a major cone phenotype that was missed in the earlier studies on α2δ4 KOs. The discrepancy could arise from a difference between human macular cones and the S-like cones studied here or be a simple sampling error because so few humans with mutations in CACNA2D4 have been described in the literature to date.

The major limitation of using Conefull: Cav1.4 mutants are inherent in the Nrl KO parent strain. The absence of rods is likely to impact the use of oxygen and metabolites in the retina, there is loss of trophic support [i.e., the absence of RdCVF (Lorentz et al., 2006; Wifvat et al., 2021)], and there are anatomical differences in how the photoreceptors pack and interact with Muller cells, evident from the disorganization of the outer limiting membrane discussed above. Importantly for future studies of synaptic biology, the rewiring of rod-ON bipolar cells to the cones (Strettoi et al., 2004; Puller et al., 2017) needs to be considered if using these mouse strains to analyze signal propagation through the downstream retinal circuitry. Interestingly, most of the molecular players involved in the development of rod and cone ON synapses are similar. One significant difference is in the use of the trans-synaptic adhesion proteins, ELFN1 or ELFN2 which are expressed by adult rods or cones, respectively, and are required for selective wiring to rod ON-bipolar or cone ON-bipolar dendrites (Cao et al., 2015, 2020). Yet, cones were found to express ELFN1 early in development which likely explains the ready rewiring of rod-ON bipolar dendrites in the Nrl KO and Conefull retinas. With these caveats in mind, the Conefull stains described here do have most of the major phenotypes of the corresponding Cav1.4 subunit KOs in wildtype retina so they could be useful for investigating cellular remodeling and synaptic plasticity in the retina.

## Data availability statement

The raw data supporting the conclusions of this article will be made available by the authors, without undue reservation.

## Ethics statement

The animal study was reviewed and approved by University of Iowa IACUC committee.

## Author contributions

JL contributed to conception and design of the study, collected data, analyzed data, and edited the manuscript. AK and CL collected data. SB contributed to conception and design of the study, analyzed data, and wrote the manuscript. All authors contributed to the article and approved the submitted version.

## Funding

Funding for this work was provided by the National Eye Institute (R01 EY026817 and R01 EY020542 to SB).

## Conflict of interest

The authors declare that the research was conducted in the absence of any commercial or financial relationships that could be construed as a potential conflict of interest.

## Publisher’s note

All claims expressed in this article are solely those of the authors and do not necessarily represent those of their affiliated organizations, or those of the publisher, the editors and the reviewers. Any product that may be evaluated in this article, or claim that may be made by its manufacturer, is not guaranteed or endorsed by the publisher.
